# Prehension and Perception of Size in Left Visual Neglect

**DOI:** 10.1155/2002/252405

**Published:** 2002-08-01

**Authors:** R. D. McIntosh, C. L. Pritchard, H. C. Dijkerman, A. D. Milner, R. C. Roberts

**Affiliations:** ^1^ Department of Psychology University of Durham Uk; ^2^ School of Psychology University of St. Andrews Fife UK; ^3^ Department of Psychology University of Utrecht Netherlands; ^4^ Department of Medicine University of Dundee UK

**Keywords:** visual neglect, hemimicropsia, perception, prehension, size distortion

## Abstract

Right hemisphere damaged patients with and without left visual neglect, and age-matched controls had objects of various sizes presented within left or right body hemispace. Subjects were asked to estimate the objects’ sizes or to reach out and grasp them, in order to assess visual size processing in perceptual-experiential and action-based contexts respectively. No impairments of size processing were detected in the prehension performance of the neglect patients but a generalised slowing of movement was observed, associated with an extended deceleration phase. Additionally both patient groups reached maximum grip aperture relatively later in the movement than did controls. For the estimation task it was predicted that the left visual neglect group would systematically underestimate the sizes of objects presented within left hemispace but no such abnormalities were observed. Possible reasons for this unexpected null finding are discussed.

## 1. Introduction

There is now abundant evidence that the rightward line bisection errors of left visual neglect are, at least in part, attributable to a distorted perception of horizontal extent. Specifically, stimuli presented in relatively leftward egocentric locations may be perceived as smaller than identical stimuli presented in relatively rightward locations. The methods developed to study this phenomenon include the "landmark task" [28] and various psychophysical size matching tasks. In the former, the subject is presented with a transected line and asked to point to the end of the line that lies closer to the transection mark. Critical trials occur when the transection mark actually bisects the line but a forced-choice response is required. On such trials most normal subjects respond randomly left or right but the majority of left neglect patients point leftward, indicating that they perceive the left half of the line as shorter than its rightward counterpart [6,7,27,28]. Size matching tasks follow a similar logic. Subjects are presented with horizontally aligned pairs of stimuli and asked to make relative size discriminations. Left neglect patients systematically underestimate the extent of stimuli presented in left hemispace relative to those presented on the right and this occurs whether the stimuli are horizontal lines, rectangles, circles or nonsense shapes (although less distortion is generally observed for vertically or radially oriented lines or rectangles) [10,26,29].

It has been suggested that distortions of perceived size associated with neglect reflect damage to neural systems concerned with the conscious representation and analysis of visual scenes, more closely linked with the ventral stream of visual processing than with the dorsal stream [23,24,26,29]. This theory predicts that, whilst these distortions should affect explicit size judgements, goal-directed visuomotor acts should be relatively unperturbed as these latter responses are primarily subserved by separate mechanisms within the

ISSN 0953-4180/01/02/$8.00 © 2001/2002 – IOS Press. All rights reserved dorsal stream [25]. This prediction has some empirical support. For instance, Robertson et al. [33] found that the rightward errors made by left neglect patients when pointing to the centre of a horizontal rod were substantially reduced if the instruction was simply to pick the rod up. The fact that a pointing response used to indicate an explicit spatial judgement revealed greater neglect than a more automatic prehensile act is consistent with the notion that the latter accessed a stream of visual processing relatively unaffected by size distortion.

A similar dissociation between perceptual experience and visuomotor guidance was reported by Pritchard et al. [31]. A neglect patient (EC) was asked to provide manual estimates of the size of objects presented within her left or right body hemispace (by matching her index-finger-thumb separation to the size of the targets) and to reach out and grasp the same objects. EC systematically underestimated the size of objects presented on the left relative to those presented on the right. Despite this misperception she was able to reach out and grasp the same objects with ease, showing normal scaling of grip aperture on both sides of space. EC's behaviour is consistent with the hypothesis that distortions of perceived size in neglect are independent of the mechanisms underlying visuomotor control. However, in order to substantiate this hypothesis it is important that EC's dissociated pattern of performance should generalise more widely across neglect patients. Accordingly, we have tested several further patients on the grasping and matching tasks employed by Pritchard et al. [31].

This paper reports the grasping and matching performance of a group of seven left visual neglect patients (including EC), seven right hemisphere damaged patients without neglect and ten healthy controls. The outcome is surprising in that the neglect group does not show any significant size distortion in the manual estimation task. There is thus no replication of the pattern of dissociation previously reported for EC [31]. Possible reasons for the lack of significant size distortion in the estimation task are considered in the Discussion. The visuomotor grasping task, however, remains of independent interest as it offers the opportunity for a broad analysis of the kinematics of prehension in neglect where previous kinematic studies of neglect behaviour have generally focused on simple motor tasks with no grasp component (e.g. [1,8,13,18–22]).

## 2. Methods

### 2.1. Subjects

Seven stroke patients with unilateral right hemisphere damage and left visual neglect (RHN+ group; mean age 68.6 years, SD 5.0), seven stroke patients with unilateral right hemisphere damage and no visual neglect (RHN− group; mean age 65.9 years, SD 10.5) and ten healthy controls (HC group; mean age 68.5 years, SD 5.0) were tested. All subjects were right handed and the groups were matched for age $[F(2,21) = 0.3;\ p > 0.7]$.

Unilateral right hemisphere damage was dually determined by CT scan and clinical signs. Patients were included in the RHN+ group only if they exhibited neglect on at least three of the following diagnostic tests: line crossing, star cancellation, figure copying, representational drawing [34] and line bisection (nine 20 cm horizontal lines). Inclusion in the RHN- group was determined by the absence of neglect on all of these tests. Patients additionally performed three verbal sub-tests of the WAIS-R (Information, Digit Span and Vocabulary), three performance sub-tests of the WAIS-R (Picture Completion, Block Design and Object Assembly) and the NART. Clinical and demographic data for all patients are summarised in Table 1.

The final column of Table 1 shows the scores obtained on canonical size-matching or landmark tasks. All patients bar one had one of these tasks administered close to the time of the present experiment. In the size-matching task, patients were presented with 64 pairs of stimuli (16 pairs each of horizontal lines, horizontal rectangles, circles and radial lines). For each stimulus type, six pairs were identical on left and right, five had a smaller figure on the left and five had a smaller figure on the right. Half of the patients were asked to identify the smaller figure and half the larger figure. In the landmark task, patients were presented with 64 pre-transected horizontal lines or gaps (32 of each). For each stimulus type, 16 stimuli were transected at the midpoint, eight were transected to the left of the midpoint and eight to the right. Patients were asked to indicate the larger segment on half of the trials and the smaller segment on the other half. Although the tendency to identify the left stimulus as the smaller was far stronger amongst neglect patients (mean = 72.5% "left is smaller" responses) than amongst patients without neglect (mean = 54.5% "left is smaller" responses) there was nonetheless some overlap between the groups in terms of individual scores.

Table 1
Clinical and demographic data for the two patient groups

| Group | Patient | Age /sex | Lesion site | Weeks poststroke | VFD (+/−) | Lines (L/R) | Stars (L/R) | Copy (+/−) | Draw (+/−) | Bisect (mm) | WAIS-R verbal | WAIS-R perform | NART IQ | Sizematch (%) |
|---|---|---|---|---|---|---|---|---|---|---|---|---|---|---|
| RHN+ | EC | 76/F | TO | 19&32* | + | 8/0 | 13/6 | + | + | 17.33 | 24 | 5 | 115 | 74.2 |
| | HW | 61/F | TP | 74 | + | 18/7 | 27/18 | + | + | 52.1 | 30 | 3 | – | 96.9 |
| | KB | 78/F | TP | 46 | − | 0/0 | 10/2 | + | − | 20.3 | 32 | 11 | 116 | 73.4 |
| | BC | 65/F | FS | 7 | − | 0/2 | 6/1 | + | + | −7.2 | 40 | 12 | – | 62.5 (lm) |
| | CS | 73/F | PO | 10 | + | 18/0 | 27/11 | + | − | 56.9 | 20 | 7 | 113 | 75.0 (lm) |
| | JR | 73/F | F | 13 | − | 9/1 | 18/8 | + | − | 21.1 | 20 | 8 | 96 | 53.1 (lm) |
| | LM | 64/F | FP | 41 | + | 0/0 | 22/2 | + | − | 23.77 | 30 | 9 | 116 | – |
| RHN− | GE | 53/F | BG | 12 | + | 0/0 | 4/2 | – | − | −4.7 | 26 | 24 | 116 | 35.9 |
| | GS | 49/F | FP | 5 | + | 0/0 | 0/1 | – | − | −0.8 | 18 | 13 | 100 | 51.6 |
| | JM | 76/M | P | 7 | + | 0/0 | 0/0 | – | − | 4.6 | 31 | 13 | 124 | 37.5 |
| | LC | 69/F | TP | 30 | + | 0/0 | 0/0 | – | − | 4.1 | 26 | 27 | – | 50.0 |
| | RR | 73/M | FP | 39 | − | 0/0 | 0/0 | – | − | −5.1 | 24 | 12 | 108 | 64.1 |
| | WA | 70/M | F | 11 | − | 0/0 | 2/0 | – | − | −2.6 | 26 | 15 | – | 70.3 |
| | SL | 71/M | F | 27 | − | 0/0 | 0/0 | – | − | −2.3 | 32 | 29 | 117 | 71.8 (lm) |

BG: basal ganglia; F: frontal; O: occipital; P: parietal; T: temporal, S: subcortical. VFD: visual field defect by confrontation; Lines:% targets omitted in each half of line crossing sheet; Stars:% targets omitted in each half of star cancellation sheet; Copying: presence/absence of neglect on figure copying; Draw: presence/absence of neglect on representational drawing; Bisect: mean bisection error (leftward −ve/rightward +ve); WAIS-R verbal: summed scaled scores for three verbal sub-tests (max = 57); WAIS-R perform: summed scaled scores for three performance sub-tests (max = 57); NART IQ: Premorbid IQ estimated from NART. Sizematch:% "left is smaller" responses across all size-matching stimuli (lm:% "left is smaller" responses across all landmark stimuli). Scores of 62.5 or more indicate a significant tendency ($p < 0.05$) to underestimate the left stimulus (relative to chance performance). *EC performed the grasping task 13 weeks before the estimation task (see [31] for further discussion of this point).

### 2.2. Procedure

Subjects were seated in front of a rectangular board (35 by 38 cm) on which two rigid plastic cylinders were presented, 13 cm to the left and right of centre and 27 cm in front of a central starting point located 5 cm from the near edge of the board (see Fig. 1). The board itself was flush with the near edge of the table and centred on the subjects mid-sagittal axis. The target cylinders were each 2 cm high and were of four different diameters (2, 3, 4, or 5 cm). On each trial, the two presented cylinders were matched for diameter but one was white and the other black. Within a block of 40 trials each colour and diameter of cylinder appeared five times at each location. Each subject performed two blocks of 40 trials with the fixed pseudo-random trial order of the first block reversed for the second block. In one block the subject made a grasping response and in the other an explicit manual size estimate was required (see below). Within each group the order of performance of the grasping and estimation task alternated between subjects.

Regardless of the mode of response the subject began each trial with his/her right index finger and thumb pinched together at the starting point and his/her eyes closed. The experimenter placed a pair of cylinders on the board and instructed the subject to open his/her eyes. In the grasping task the subject was required to reach out and grasp the white cylinder. In the manual estimation task the subject was asked to separate his/her finger and thumb (without reaching out) so that the aperture created matched the diameter of the white cylinder. In both tasks head and eye movements were unrestricted and no time constraints were imposed. During the matching task, the experimenter monitored head and eye movements closely in order to ensure that subjects responded exclusively to the white cylinder as instructed.

### 2.3. Data collection

Data was collected using either an opto-electronic movement analysis system (Optotrak 3020: Northern Digital Inc. Waterloo, Canada) or a portable electromagnetic movement analysis system (Minibird, Ascension Technology). Infrared or magnetic markers, depending on the system used, were attached to the distal phalanxes of the subject's right index finger and thumb. The 3D positions of these markers were sampled at a rate of 100 Hz (Optotrak) or 86.1 Hz (Minibird) throughout the movement (grasping task) or for one second after the subject was satisfied that his or her finger-thumb aperture matched the diameter of the white cylinder (estimation task). The Optotrak system was used for eight of the control subjects, six of the RHN− patients and three of the RHN+ patients. The

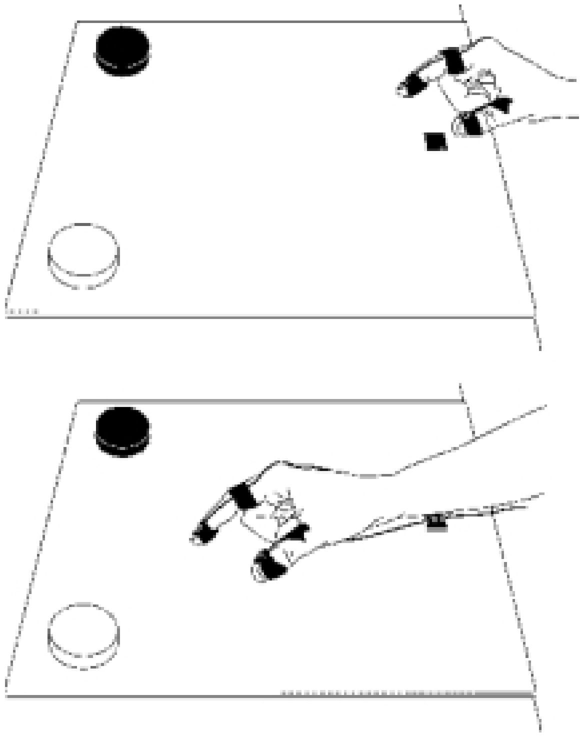

Fig. 1. (Upper) In the estimation task the subject separated his/her finger and thumb (without reaching out) to indicate the diameter of the white cylinder. (Lower) In the grasping task the subject reached out and grasped the white cylinder. In both tasks, the size and relative positions of white and black cylinders varied from trial to trial.

other subjects were unable to travel to St. Andrews and the portable Minibird system was used for data collection.

Because of the use of two different recording systems, a control experiment was carried out to assess whether any biases might be introduced to the kinematic data according to the system used. Six healthy subjects performed a prehension task similar to that of the main study. Each subject performed two blocks of 48 grasping responses to objects of different sizes and at various distances on either side of the body midline. The Optotrak system was used to record responses in one block and the Minibird system was used in the other (the order of blocks was alternated between subjects). The kinematic variables examined were the same as for the main experiment (see next section). For each kinematic variable, a repeated-measures ANOVA by system (Optotrak, Minibird), side (left, right) and size (20, 50 mm) was performed. The patterns of data obtained from the two recording systems were closely comparable. Across all dependent measures, only one significant effect involving the factor of system was found. This was a main effect of system on Maximum Grip Aperture (MGA) [$F(1,5) = 68.1$; $p < 0.0005$], reflecting the larger MGAs recorded by the Minibird system (means: 76.3 mm and 61.6 mm for Minibird and Optotrak respectively ). This was due to differences between the size of Optotrak and Minibird markers and between the positions of their optimal placement on the hand. This constant bias is unimportant with respect to the main experiment, however, since the hypotheses to be tested do not relate to the size of MGA per se but rather to its relationship with object size and side of stimulus presentation.

### *2.4. Data analysis*

The data were filtered using a 10 Hz Butterworth dual-pass filter. For the estimation task the mean vectored separation of the two markers during the one second recording period was computed. For the grasping task movement onset and offset were determined by comparison of the tangential velocity of the hand against a threshold value (50 mm/s) and only data from within this time window were analysed. Velocity was computed from the thumb whilst the vectored separation of finger and thumb markers was used to characterise the grasp component. The following kinematic variables were examined: Maximum Grip Aperture (MGA), Movement Time (MT), Peak Velocity (PV), Time to Peak Velocity (TPV), Time After Peak Velocity (TAPV) and Percentage Time to MGA [= (Time to MGA/MT)*100].

The manual estimation apertures and the six kinematic variables for the grasping task were submitted to separate repeated measures ANOVAs with group (HC, RHN−, RHN+) as a between-subjects factor and side of presentation (left, right) and object size (2, 3, 4, 5 cm) as within-subjects factors with Huynh-Feldt adjustments ($\Sigma$) to the degrees of freedom.

## 3. Results

### *3.1. Estimation task*

Figure 2 displays manual estimation aperture as a function of side and object size for each group. ANOVA revealed a significant main effect of size [$\Sigma = 0.5$; $F(1.4, 29.9) = 298.0$; $p < 0.001$] and a just significant three-way interaction of side by size by group [$F(6) = 2.4$; $p < 0.05$]. This interaction was explored using separate repeated-measures

ANOVAs by side and size for each group but no reliable two-way interactions were found. Notably, the apparent trend toward a side by size interaction in the RHN+ group (Fig. 2) did not approach significance [$\Sigma = 0.5$; $F(1.6, 9.4) = 2.4$; $p > 0.15$]. The crucial finding, therefore, was of no lateralised differences in the estimation of object size for any subject group.

This result is surprising given the starting hypotheses. However, it may be worth recalling that size underestimation on canonical screening tasks was imperfectly associated with neglect on other clinical tasks (see Table 1). An alternative subdivision of the patient population was thus created in order to examine more closely whether size-underestimation on canonical tasks was predictive of performance on the manual estimation task. A repeated measures ANOVA by side and object size was performed on the manual responses of those eight patients who showed significant left-sided underestimation on size-matching or landmark screening tasks (see Table 1). Even for this patient group, side of presentation did not influence manual estimation aperture [$F(1, 7) = 0.6$; $p > 0.45$] and did not interact with object size [$\Sigma = 0.4$; $F(2.4, 16.5) = 0.8$; $p > 0.45$]. Accordingly, performance on canonical tests of size distortion, regardless of clinical neglect, was not predictive of manual estimation performance.

## 4. Grasping task

*Maximum Grip Aperture* (*MGA*). Figure 3 shows MGA as a function of object size and side of response for each subject group. ANOVA confirmed the expected effect of object size [$\Sigma = 0.5$; $F(1.5, 30.6) = 217.5$; $p < 0.0005$]. No other effects reached significance though there was a non-significant tendency for MGA to be smaller on the left [$F(1, 21) = 4.1$; $p < 0.1$]. This tendency has been observed previously and attributed to biomechanical factors differentially affecting ipsilateral and contralateral reach-to-grasp movements [3,30,31]. The factor of side did not interact with subject group [$F(2, 21) = 0.1$; $p > 0.9$].

*Movement Time* (*MT*). Figure 4 shows MT as a function of side and group. ANOVA found significant main effects of side [$F(1, 21) = 56.1$; $p < 0.0005$] and group [$F(2, 21) = 4.6$; $p < 0.05$] but no reliable interaction between these factors [$F(2, 21) = 2.6$; $p = 0.1$]. Planned comparisons confirmed longer movements in the RHN+ group compared with the HC subjects ($p < 0.01$) but not compared with the RHN− group.

*Peak Velocity* (*PV*). The pattern of PV (Fig. 5) corresponded closely to that of MT, with significant effects of side [$F(1, 21) = 47.3$; $p < 0.0005$] and group [$F(2, 21) = 4.1$; $p < 0.05$]. Planned comparisons found RHN+ patients to be reliably slower than HC subjects ($p < 0.01$).

*Time to Peak Velocity* (*TPV*) *and Time After Peak Velocity* (*TAPV*). The analysis of TPV found a main effect of side only [$F(1, 21) = 56.4$; $p < 0.0005$]. All groups reached peak velocity more rapidly on the right consistent with their shorter movement times to this location. Figure 6 shows TPV and TAPV as a function of side and group. In contrast to the lack of difference between groups in the time taken to reach peak velocity, the time spent moving thereafter was much greater for neglect patients. This was confirmed by a significant main effect of group [$F(2, 21) = 8.3$; $p < 0.005$] with planned comparisons identifying reliable differences between RHN+ patients and HC subjects ($p < 0.005$) and between the two patient groups ($p < 0.05$). A just significant interaction between subject group and side of response was also found [$F(2, 21) = 4.4$; $p < 0.05$]. Visual inspection of Fig. 6 suggests that this may reflect the longer deceleration times of neglect patients when responding to the left. However, an ANOVA by side and size conducted on the neglect group's data did not find the effect of side to be reliable [$F(1, 6) = 4.7$; $p < 0.1$].

*Percentage time to MGA* (*%TMGA*). The analysis of %TMGA found main effects of object size [$\Sigma = 0.8$; $F(2.4, 49.8) = 6.0$; $p < 0.005$] and subject group [$F(2, 21) = 7.7$; $p < 0.005$] but not side of response (Fig. 8). Planned comparisons identified reliable differences between the HC and RHN+ groups ($p < 0.005$) and between the HC and RHN− groups ($p < 0.05$). MGA thus occurred relatively late in patients with right hemisphere damage regardless of neglect.

In summary, all groups took longer to execute leftward than rightward movements, reaching a higher peak velocity at an earlier time when reaching to grasp objects in right body hemispace (the ipsilateral side with respect to the responding arm) (see also [4]). Neglect patients were abnormally slow to execute prehension movements and this was largely attributable to a prolonged period of movement following peak velocity. Additionally, right hemisphere damage was found to lead to a relative delay in attaining maximum grip aperture.

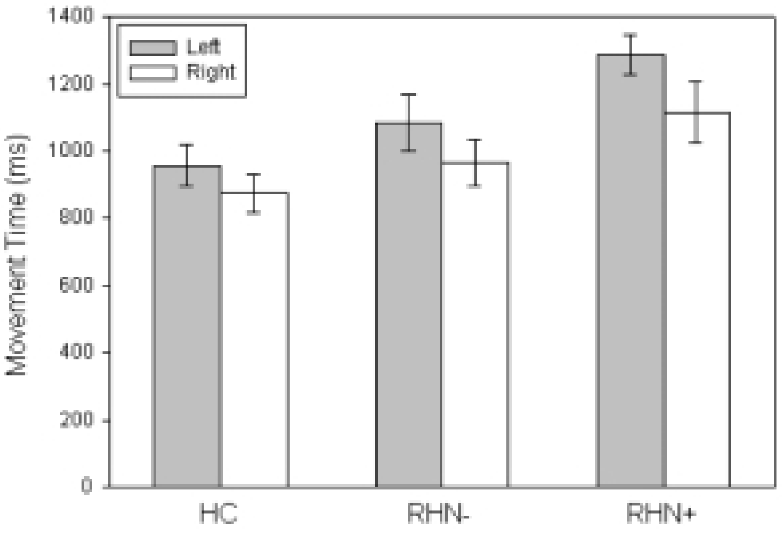

Fig. 4. Movement Time as a function of side of presentation and subject group (2 SE indicated).

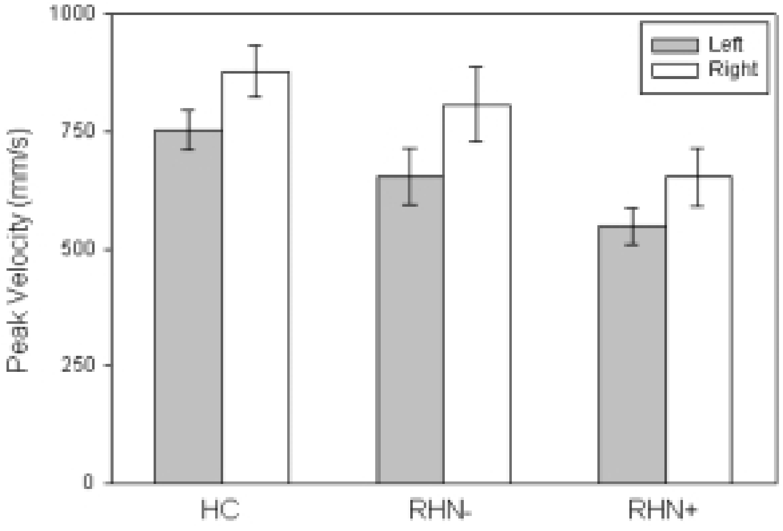

Fig. 5. Peak Velocity as a function of side of presentation and subject group (2 SE indicated).

## 5. Discussion

The primary purpose of this study was to investigate whether the pattern of dissociated performance between manual size estimation and visuomotor grip scaling observed for patient EC [31] could be replicated across a wider group of left visual neglect patients. However, this study failed to replicate the more basic finding of size underestimation for objects in left hemispace. Thus, although the neglect group did not underscale their grip when grasping objects located in left hemispace, this preservation of normal size processing in a visuomotor context was not divergent from perceptual estimates of object size. The most salient general feature of the present results is the absence of lateralised abnormalities amongst neglect patients in both the estimation and the grasping tasks.

### 5.1. Estimation task

For brevity, the present paper has not reported individual subjects' data but it should be noted that the symmetry of size estimation apparent in the group results was representative of all neglect patients with the

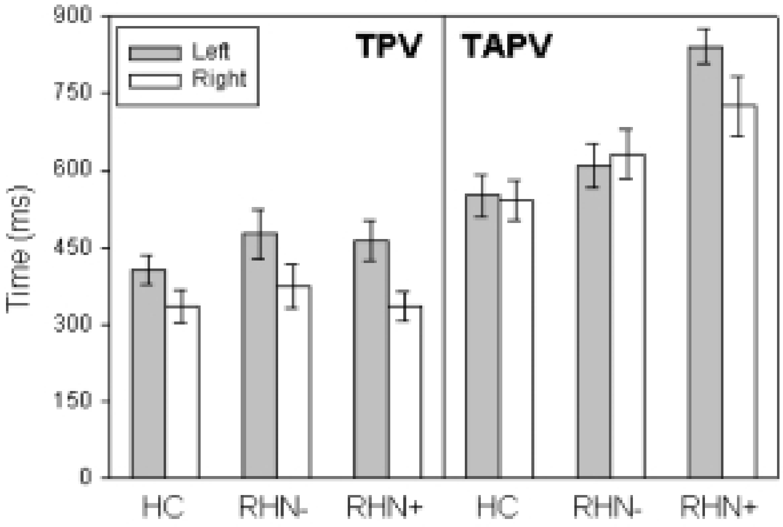

Fig. 6. Time to Peak Velocity (TPV) and Time After Peak Velocity (TAPV) as a function of side and subject group (2 SE indicated).

sole exception of EC, the performance of whom has been fully documented by Pritchard et al. [31]. Therefore, the twin mysteries to be explained with regard to the manual estimation task are (a) why did the neglect group fail to show significant lateralised size distortion and (b) why was patient EC an exception to this general rule?

The failure to observe significant size distortion effects in the manual estimation task cannot be attributed simply to a low prevalence of this symptom amongst the neglect patients studied (see Table 1). Moreover, even a preselected group of patients who each showed significant underestimation of the left stimulus on canonical screening tasks did not exhibit any such tendency on the manual estimation task. Therefore, it must be assumed that the manual estimation task employed is somehow unsuited to exposing the kind of perceptual distortion that neglect patients typically experience (and further that EC is, in some manner, an atypical neglect patient). There are several differences between the manual estimation task and the tasks used in previous studies that might be considered relevant in this regard. One obvious difference is that the present study employed solid target objects rather than the two-dimensional pictorial stimuli favoured in previous work [6,7,10,15,26,28,29]. However, not only is there no clear reason to expect size distortion to be restricted to pictorial stimuli but our recent observations indicate that robust lateralised size distortion can be observed in neglect patients asked to make relative judgements about the horizontal extent of solid rectangular objects (unpublished data).

A more promising account relates to the fact that the target objects were cylindrical and thus circular in cross-section. (Cylindrical stimuli were chosen so that hand orientation would not be too constrained during grasping, allowing subjects to respond in the most comfortable manner; of course, an equivalent degree of flexibility will have been available to subjects performing the estimation task.) When asked to estimate manually the size of the target cylinder the subject might elect to estimate its lateral diameter, its radial (depth) diameter or its diameter at any intermediate orientation. The orientation chosen may be relevant because the size distortion exhibited in neglect seems predominantly to affect the lateral axis [10,26,29]. A patient choosing to match a cylinder's diameter front-to-back might thus be less prone to exhibit any abnormality of size processing. On this account the lack of a lateral vector in the dimension of the object to which the patients attended during size estimation might underlie the lack of observable neglect on this task. Circumstantial evidence for this proposal can be found in the manual estimation data. Analysis of the responses made by neglect patients reveals that the mean absolute angle formed by the finger-thumb aperture with respect to the depth axis, in the horizontal plane, was 18.7° (SD 10.1). The minimal representation of the lateral axis in these estimation responses is consistent with the proposal that the patients may have been attending primarily to the radial diameter of the target cylinders.

The above account seems plausible but there is good reason to believe it cannot explain fully the lack of size-underestimation phenomena observed in manual esti-

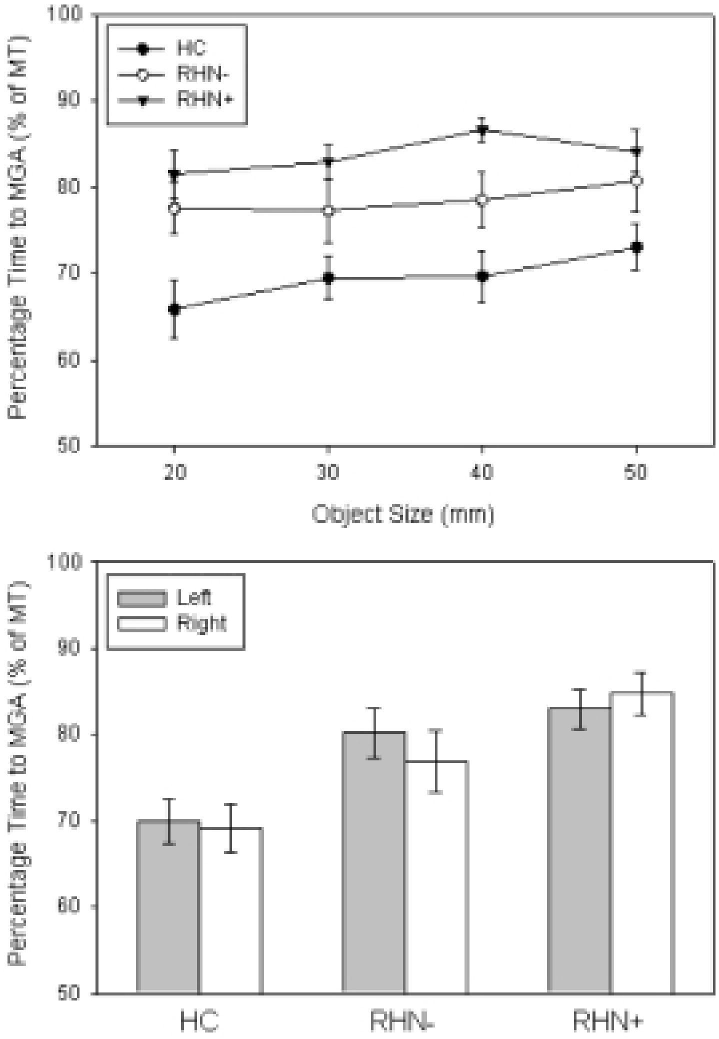

Fig. 7. (Upper) Percentage Time to Maximum Grip Aperture as a function of object size and subject group (2 SE indicated). (Lower) Percentage Time to Maximum Grip Aperture as a function of side and subject group (2 SE indicated).

mation. Specifically, the three neglect patients tested on the conventional (2D) size-matching task (EC, HW, KB) all showed a perceptual underscaling of the leftward stimulus that was as pronounced for radially oriented lines (and for circles) as for horizontally oriented lines and rectangles. This result is somewhat surprising in light of previous evidence that radial size distortion is relatively uncommon in neglect [10,26,29]. Nonetheless, it indicates that (at least) three members of the neglect group experienced size distortions that were (at least) bi-dimensional, affecting both lateral and radial axes. Such patients would be expected to underestimate left-sided targets in the estimation task regardless of the orientation of the attended diameter of the target cylinder. Accordingly, the use of cylindrical stimuli cannot have been the only factor underlying the failure to observe significant size distortion in the estimation task. Even so, it is worth remarking that future studies of size estimation in neglect would be well advised to control explicitly the stimulus dimension to which subjects respond.

One final explanation for the absence of significant size distortion will be advanced. On this account, the crucial distinction between the estimation task and standard assessments of size distortion may lie in the type of judgement required rather than in the specific form of the target objects. Whilst previous studies have asked subjects to make relative size judgements concerning (at least) two simultaneously presented targets [6,7,10, 26–29], the manual estimation task requires the subject to indicate the absolute size of a single object (two cylinders were presented on each trial, but the subject was instructed to attend only to the white one). Such procedural differences might modify the expression of size distortion phenomena. In particular, if a tendency to underscale leftward extents results from a lack of attention to stimuli in leftward locations, as already suggested by several authors (e.g. [6,10,15,27, 32]), then the presence of competing task-relevant stimulation from relatively rightward locations may be a precondition for this bias to be expressed. Consistent with this, Kerkhoff has recently reported that size distortion in neglect is ameliorated if the stimuli to be compared are presented sequentially rather than simultaneously [15]. Like many other symptoms associated with visual neglect, lateralised size distortion may be a relative and not an absolute phenomenon [16,17].

If this final explanation is correct then neglect patients' estimates of the absolute sizes of single targets should be independent of the location of target presentation, and the left-sided size underestimation exhibited by EC on the manual estimation task [31] would require some origin other than visual neglect. It is suggested that EC's peculiar pattern of impairment might instead be related to the occipito-temporal focus of her lesion which, whilst unusual amongst neglect patients, has been strongly implicated in the genesis of hemimicropsia [2,5,14]. If so, the behavioural dissociation originally reported by Pritchard et al. [31] would remain of relevance to the neural organisation of visual size processing but would be coincidental with regard to EC's visual neglect. It is unfortunate that EC is not available for further testing but the present findings compel the conclusion that her pattern of performance is not generally representative of neglect impairment.

### 5.2. *Grasping task*

In the grasping task, no lateralised differences of size processing were found in the neglect group though the full significance of this result is unclear given that it does not contrast with any demonstrated abnormality of explicit size estimation. The observed normality of grip scaling might indicate that size processing for action proceeds normally in neglect and is not subject to lateralised distortions. On the other hand, this apparent normality could be related to the use of cylindrical stimuli and/or some other methodological aspect of the grasping task employed, as already discussed in relation to the estimation task. However, although the grasping task was designed primarily to investigate the calibration of grip aperture, several additional variables were studied allowing for a more broad description of the prehension movements performed. As already noted, the most salient outcome was the absence of clear direction-specific impairments of velocity control, grasp formation and grasp-transport coupling in the patient groups. This pattern supports Konczak and Karnath's contention that visual neglect does not characteristically alter the kinematics of goal-directed movements in a lateralised manner [13,18]. Nonetheless, several pronounced non-lateralised abnormalities emerged which may be of wider relevance to the control of prehension.

With respect to the transport component, there was a generalised bradykinesia in the neglect group, associated with a lower peak velocity and an extended final phase of movement after peak velocity was reached. Generalised bradykinesia has been observed in neglect patients across a range of simple motor tasks (e.g. [1, 3,18–21]) so it is unsurprising that it is also apparent in prehension. However, although these abnormalities of transport were statistically significant in the neglect group only, the same trends were qualitatively present in the right hemisphere damaged patients without neglect. This suggests that they are not specific to neglect but may instead be related to the extent and/or locus of brain damage. Consistent with this view, several authors have found the movement times of right hemisphere damaged patients without neglect to be intermediate with respect to neglect patients and controls, and some have observed a statistically significant degree of slowing in such patients [18,19].

At least two previous studies have linked increased movement times in right hemisphere damaged patients with a prolongation of movement after peak velocity [9,35]. Winstein and Pohl [35] found that this pattern became more pronounced as the accuracy demands of aiming movements increased but they observed no comparable effects in left hemisphere damaged patients, who tended to prolong all phases of movement whilst preserving the normal temporal proportions (see also [9]). This was interpreted as evidence that the right hemisphere is preferentially involved in executing the rapid closed-loop adjustments that guide the hand as it nears its target. The findings of the present study are consistent with this hypothesis. The use of a variety of object sizes may have limited the scope for the adoption of stereotyped responses and emphasised the requirement for precise on-line control, forcing the right hemisphere damaged patients, in whom this ability is compromised, to prolong the period during which closedloop adjustments are made. Moreover, this requirement for a prolongation of the movement may contribute to the tendency for the patient group to move more slowly (i.e. with a lower peak velocity) than normal subjects.

Another notable finding was that right hemisphere damaged patients showed delayed grasp formation, reaching maximum grip aperture relatively late in the movement. This abnormality was independent of hemispace and was present in both patient groups (though it was slightly more pronounced in neglect patients). Similar results have been reported by Hermsdörfer et al. [9] who studied the prehension characteristics of right and left hemisphere damaged patients across a range of object sizes comparable to that used in the present study. In addition to a prolonged phase of transport after peak velocity (see above), these authors found that maximum grip aperture was relatively delayed in the right hemisphere damaged group. This pattern is again consistent with the notion that right hemisphere damage compromises the utilisation of visual feedback from movement [35]. However, it is somewhat unclear whether prolongation of hand transport and retardation of grip formation represent separable impairments of transport and grasp control or whether the former occurs to accommodate the latter. In the present study, Figs 6 and 7 hint at some degree of independence since, relative to patients without neglect, neglect patients were differentially more impaired in transport than in grasp parameters. This inference, though speculative, is consistent with Jeannerod's influential hypothesis of distinct "visuomotor channels" for the transport and grasp components of prehension [11,12].

### 5.3. Conclusion

Pritchard et al. [31] reported that neglect patient EC systematically underestimated the size of cylinders presented in left hemispace but scaled her grip appropriately when reaching out to pick them up. The primary purpose of the present study was to investigate whether this pattern of dissociation could be observed more widely in a group of left visual neglect patients. However, the neglect group did not demonstrate any manual size underestimation of objects presented in left hemispace. This failure to replicate EC's pattern of performance suggests that her lateralised asymmetry of manual size estimation was not a consequence of her neglect. It may instead have been due to hemimicropsia, a relatively little studied clinical condition associated with lesions of the occipito-temporal junction [2, 5,14]. To substantiate this hypothesis it will be important to ascertain whether EC's pattern of preserved visuomotor size processing with impaired manual size estimation can be replicated in further patients with lateralised micropsia (or macropsia) of cortical origin.

A conjectural explanation has been advanced for the present failure to observe significant size distortion in the manual estimation task. It has been proposed that the size distortions associated with neglect may require the presence of competing stimulation from relatively left and rightward locations. That is, patients may under-scale leftward extents only when they must compare them with relatively rightward extents (as in size matching, landmark, line bisection and line extension tasks). This implies that such distortions may arise only under a rather restricted range of experimental conditions and is a less pervasive phenomenon than has previously been assumed. We are now evaluating this proposal through a series of studies investigating the influence of attentional competition and mode of response on the expression of size distortion in neglect. These studies will hopefully shed further light on the functional significance of this perplexing aspect of the neglect syndrome.

## Acknowledgements

This work was supported by a grant from the Leverhulme trust to ADM and HCD and a Wellcome Trust grant to ADM. The authors are grateful to Kevin McClements for help with data collection, to Roger Newport for technical advice and to Yves Rossetti for comments on an earlier version of this manuscript.

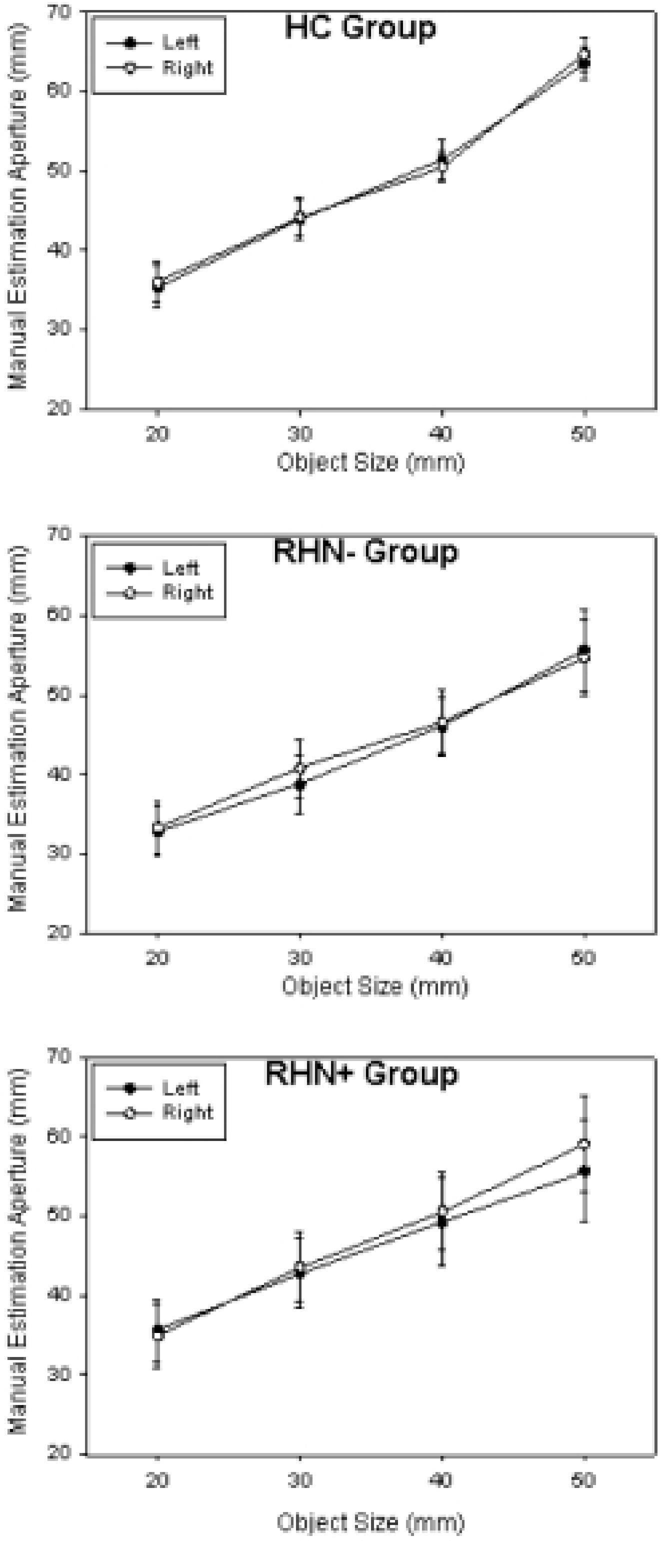

Fig. 2. Manual estimation aperture as a function of side and object size for each subject group (2 SE indicated).

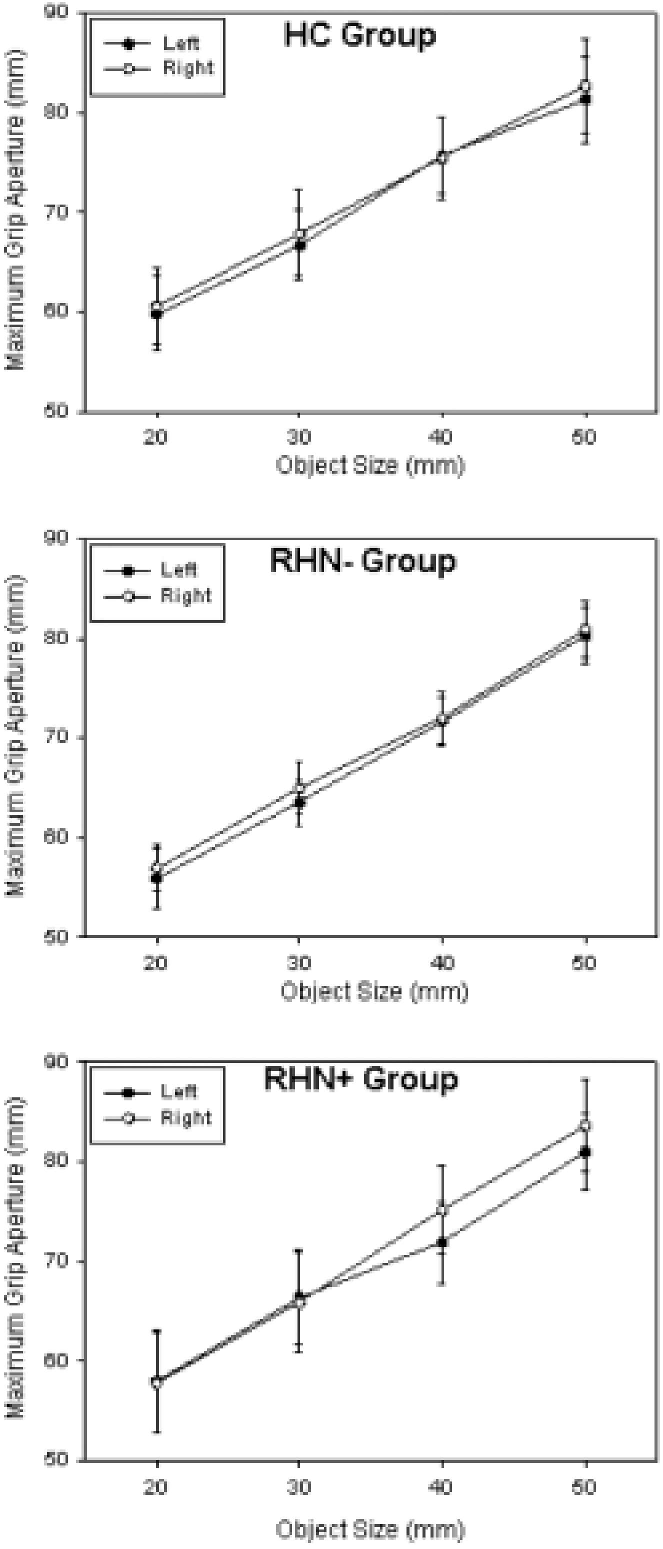

Fig. 3. Maximum Grip Aperture as a function of side and object size for each subject group (2 SE indicated).
